# Neutrophil Extracellular Traps: Signaling Properties and Disease Relevance

**DOI:** 10.1155/2020/9254087

**Published:** 2020-07-28

**Authors:** Tiewei Li, Zhengyan Zhang, Xiaojuan Li, Geng Dong, Min Zhang, Zhe Xu, Junmei Yang

**Affiliations:** ^1^Zhengzhou Key Laboratory of Children's Infection and Immunity, Children's Hospital Affiliated to Zhengzhou University, Henan Children's Hospital, Zhengzhou Children's Hospital, 33 Longhu Waihuan East Street, Jinshui District, Zhengzhou 450000, China; ^2^Children's Hospital Affiliated to Zhengzhou University, Henan Children's Hospital, Zhengzhou Children's Hospital, 33 Longhu Waihuan East Street, Jinshui District, Zhengzhou 450000, China

## Abstract

Neutrophil extracellular traps (NETs) are characterized as extracellular DNA fibers comprised of histone and cytoplasmic granule proteins. NETs were first described as a form of innate response against pathogen invasion, which can capture pathogens, degrade bacterial toxic factors, and kill bacteria. Additionally, NETs also provide a scaffold for protein and cell binding. Protein binding to NETs further activate the coagulation system which participates in thrombosis. In addition, NETs also can damage the tissues due to the proteins they carry. Many studies have suggested that the excessive formation of NETs may contribute to a range of diseases, including thrombosis, atherosclerosis, autoimmune diseases, and sepsis. In this review, we describe the structure and components of NETs, models of NET formation, and detection methods. We also discuss the molecular mechanism of NET formation and their disease relevance. Modulation of NET formation may provide a new route for the prevention and treatment of releated human diseases.

## 1. Introduction

Neutrophils are the most abundant type of white blood cells in peripheral blood and participate in many physiological and pathological processes of the body [1]. Neutrophils play an important role in the immune system's first line of defense against bacterial and viral infection through their phagocytosis and the activity of intracellular proteins [2]. In addition, neutrophils can also release neutrophil extracellular traps (NETs) under pathological conditions or in vitro stimulation. The structure of NETs released from neutrophils under phorbol myristate acetate (PMA) or lipopolysaccharide (LPS) stimulation was first observed by Brinkmann et al. in 2004 using electron microscopy [3]. NETs carry cytoplasmic and granular antimicrobial proteins which play an important role in host defense. In sepsis, neutrophils invade and accumulate at the infected sites and can be induced to release NETs involved in the development of sepsis [4]. NETs also provide a scaffold for the binding of platelets, red blood cells, and the plasma proteins [5]. The proteins binding to NETs further activate both the cellular and plasmatic coagulation system [6, 7]. In addition, NETs are also involved in the formation and development of atherosclerotic plaques, and treatment with deoxyribonuclease I (DNase I) significantly reduces the plaque size in experimental models [8]. In addition, NET-associated proteins may participate in the pathogenesis of autoimmune diseases by inducing the body to generate autoantibodies against autoantigens [9]. Studies also demonstrated that NETs play an important role in diabetes [10, 11], Alzheimer's disease [12], and tumor progression [13, 14]. NETs may also occlude glandular ducts as seen in pancreatitis [15]. In this review, we will mainly focus on the molecular mechanisms by which NETs are formed and the relationship between NETs and thrombosis, atherosclerosis, autoimmune diseases, and sepsis.

## 2. Structural Components, Detection, and Formation of NETs

### 2.1. Structural Components of NETs

NETs are extracellular traps mainly composed of DNA, and treatment with DNase can significantly disrupt the NETs structure [16]. Scanning electron microscopy has shown that the diameter of DNA filaments is 15–17 nm, and many spherical substances with a diameter of about 25 nm, which are likely to be proteins, bind to the DNA. Proteins binding to NETs include histones H1, H2A, H2B, H3, and H4 and neutrophil elastase (NE) [3]. Meanwhile, a variety of proteins have been found on NETs by mass spectrometry, such as myeloperoxidase (MPO), cathepsin G, neutrophil defensins, and the cytoplasmic calprotectin protein complex (also called S100A8/A9). The core proteins of NETs are H2A, H2B, H3, and H4, which account for about 70% of the total NET proteins, followed by NE, S100A8, lactotransferrin, azurocidin, and cathepsin G [16, 17]. In addition, previous studies and our work also found that NETs also contain matrix metalloproteinase-9 (MMP-9), [18, 19], tissue factor (TF) [20], von Willebrand factor (vWF), and fibrinogen [5, 21].

### 2.2. Detection of NETs

There are two methods available for identifying NETs. One method involves staining secreted DNA with SYTOX Green nucleic acid dye, which can be further observed by fluorescence microscope, and fluorescence intensity can be detected with a microplate reader. This method is simple and direct, but only useful under select circumstances. The second method involves detecting the specific components of NETs, including DNA, citrullinated histone H3 (citH3), and MPO (or NE), by immunofluorescence. Therefore, in cells or tissues, the immunofluorescence detection of NETs is a combination of DNA+citH3+MPO/NE (Table 1) [5, 22, 23].

In peripheral blood, various components carried on NETs are analyzed, leading to large variations of reports on NET detection targets. Nucleosomes, dsDNA, MPO-DNA complexes, and citrullinated histone H4 (citH4) have been reported to be markers of cell death and NETs in vivo [24]. Aldabbous et al. [22] detected dsDNA, MPO, NE, and MPO-DNA complexes in peripheral blood and pointed out that the level of NETs increased when the levels of at least two of the four NET components were increased. However, some studies only detected dsDNA [25, 26], which is fast and simple, but less specific. By comprehensively considering the structural components of NETs and previous studies, the detection of NETs in blood can be summarized as a combination of the detection of the following factors (Table 1): dsDNA, nucleosomes, MPO-DNA complexes (MPO or NE), and citH3 (citH4).

### 2.3. The Formation of NETs

NETs are released by neutrophils in different forms depending on the specific assay employed and the stimuli used. At present, at least three NET formation pathways have been identified, namely, vesicle-mediated, cell lytic, and mitochondrial release (Table 2) [27]. The analysis of NET formation in vitro is complicated by the fact that neutrophils respond rather uniformly to strong NET instigators like PMA and ionomycin, but only a limited amount of neutrophils undergoes NET formation in response to bacteria, fungi, and LPS leading to the presence of both unstimulated neutrophils and NETs in the same well [28]. The following hypotheses of NET formation have been proposed:

1. Vesicle-mediated NET releases: under external stimulation with *Staphylococcus aureus*, the lobular nucleus of the neutrophil becomes round, and chromatin is uniformly concentrated in the nucleus. The nuclear membrane is then ruptured, and the vesicles wrapped with DNA proposedly move to the cell membrane. These vesicles subsequently fuse with the cell membrane and release the DNA in the vesicles, i.e., release NETs. Generally, NETs can be released within 30–60 min via vesicles [29]
2. Cell lytic: prototypical formation of NETs in response to PMA leads to cell lysis and requires more time (3–4 h). The nuclear membrane is enzymatically degraded upon external stimulation. Study reported that NE and MPO reportedly cooperate in nuclear lysis and chromatin decondensation in PMA-induced NET formation [30]
3. Mitochondrial DNA: currently, few studies have reported that neutrophils release mitochondrial DNA to form NETs in a reactive oxygen species- (ROS-) dependent manner, while the detailed molecular mechanisms of mitochondrial DNA release are still unclear [31–33].

## 3. Signaling Mechanisms of NET Formation

### 3.1. PAD4

**Figure 1:**
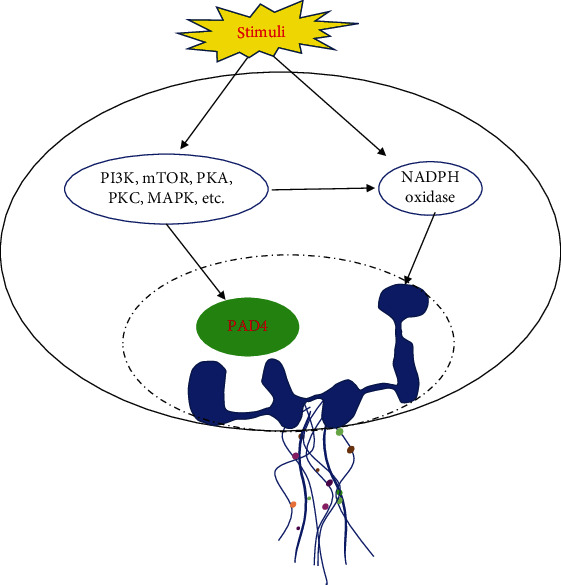
The mechanisms of NETs formation. Under the different stimulations, neutrophils release NETs via different signaling molecules.

NETs, as a weapon for neutrophils to function, have received a lot of attention and, in recent years, have been the subject of intense investigation in the field of immunology. Detailed molecular mechanisms regulating NET formation have received significant attention, and some progress has been made. Multiple studies have reported that PAD4, which mediate histone citrullination, plays an important role in the formation of NET, to form NETs [34–37]. PAD4 was first identified during the differentiation of HL-60 cells into granulocytes [38] and is highly expressed in human peripheral blood neutrophils [39]. Inhibition of PAD4 attenuates the citrullination of histones H3 and H4 and inhibits NET formation [40]. We have found that PAD4 is required for lysophosphatidic acid- (LPA-) induced histone H3 citrullination and NET formation. Lewis et al. [41] further confirmed that PAD4 is involved in the formation of NETs induced by calcium ion carriers in mouse neutrophils. Deletion of the *PAD4* gene renders neutrophils unable to form NETs under external infection or stimulation [36, 42, 43]. In addition, studies reported that NETs also could be induced by a variety of stimulators through PAD4 signaling, such as calcium antagonists, tumor necrosis factor alpha (TNF-*α*), N-formyl-L-methionyl-L-leucyl-phenylalanine (fMLP), LPS, and H_2_O_2_ (Figure 1) [44]. Citrullination of histones leads to a loss of charge and alters DNA and protein-binding properties of histones favoring chromatin decondensation [38]. Moreover, citrullination has recently been shown to impact proteolysis: Tilvawala et al. [45] demonstrated enhances proteolysis by serine proteases via PAD4-mediated inhibition of serine protease inhibitors. Moreover, proteolysis by calpain is enhanced by conformational changes induced by citrullination thus favoring nuclear lamina breakdown and chromatin decondensation [46].

### 3.2. NADPH

Other signaling pathways can also induce the formation of NETs. Douda et al. [47] reported that PMA induced NET formation via a nicotinamide adenine dinucleotide phosphate- (NADPH-) dependent signaling pathway, rather than via PAD4. Blocking NADPH significantly inhibits the formation of PMA-induced NETs [48]. Compared with the importance of ROS generated by the NADPH oxidase in the process of PMA-induced NET formation, inhibition of ROS only partially inhibits NET formation induced by *Candida albicans* and Gram-positive group B *Streptococcus* [49]. Neeli et al. [50] reported that NADPH was also required for LPS-induced histone citrullination and NET formation, suggesting that NADPH might participate in the regulation of NET formation through PAD4. In addition, many stimulators depend on the NADPH signaling pathway to induce NET formation, such as the calcium ion carrier A23187 [47], *Pseudomonas aeruginosa* [48], and oxidized low-density lipoprotein (LDL) [51] (Figure 1).

### 3.3. Other Signaling Molecules

Marcos et al. [23] reported that CXCL8/CXCL2-induced NET formation is independent of the NADPH signaling pathway but relies on Src and MAPK signaling pathways. In addition, many signaling molecules could regulate NET formation, such as mammalian target of rapamycin (mTOR), protein kinase C (PKC), and protein kinase A (PKA). McInturff et al. [52] reported that the mTOR inhibitor rapamycin inhibited LPS-induced NET through regulating HIF-1*α*. mTOR also induces autophagy to promote fMLP-induced NET formation [53]. The above findings indicate that the downstream signaling cascades triggered by the same signal molecule are also different in the process of NET formation under different stimuli. There are also many regulatory molecules in the pathway upstream of NADPH. Blocking PKC and Raf-MEK-ERK significantly inhibits PMA-induced ROS production and NET formation [54]. In addition, different PKC subtypes have different effects on inducing histone citrullination: PKA*α* inhibits histone citrullination and the formation of NETs, while PKC*ζ* promotes citrullination of histones and NET formation [55]. Studies have demonstrated that PI3K [56], Rac [57], TLR [58], and the Fc*γ*RIIIB receptor [59] participate in the formation process of NETs. Kenny et al. [49] further demonstrated that the signaling mechanisms regulating NET formation were different depending on the different stimulants, indicating that the regulatory mechanism of NET formation is not a uniform process, which requires further investigation (Figure 1).

## 4. NETs and Thrombosis

### 4.1. Influence of NETs on Coagulation System

In recent years, the role of NETs in thrombosis has attracted a lot of attention in both clinical and basic research. Studies have demonstrated that NETs can provide a scaffold for the binding of fibronectin, fibrinogen, vWF, and other protein components involved in thrombosis. NETs also trap red blood cells, promote platelet aggregation, and induce the formation of thrombi [5]. Many of the components on NETs participate in the coagulation process directly or indirectly [6]. For example, MPO and neutrophil serine proteases can inactivate anticoagulants, such as TF pathway inhibitors (TFPI) [60] and thrombomodulin [61]. NETs also contain TF, which is considered to be a main initiator of the extrinsic pathway of plasmatic coagulation in vivo [20, 62–64]. TF can activate intrinsic pathways and cause the formation of a large amounts of fibrin in the blood vessels [65]. It induces the generation of thrombin and promotes the coagulation process [20, 66, 67]. In addition, NETs lead to the formation FXIIa, and thus intrinsic pathway of plasmatic coagulation [66]. Gould et al. [68] confirmed that NETs activated the coagulation system through extrinsic pathways in platelet-depleted plasma and increased the risk of coagulation by inducing thrombin generation in platelet-rich plasma. Depletion of NETs by DNase further enhances thrombin generation [68], suggesting that degradation of NETs might release the procoagulant factors. However, Noubouossie et al. [69] found that purified human neutrophil DNA and histones significantly induce thrombin generation, while complete NETs and citrullinated histones have no effect on thrombin generation. At present, the effects of NETs on the coagulation system are still controversial, possibly due to the use of the different experimental methods. First, the functions of isolated NET components and those carried by activated neutrophils may be different. Second, animal experiments are affected by more factors than the in vitro experiments. In vivo, NETs can disrupt blood flow, damage endothelia, and promote platelet aggregation [5, 66, 70]. Meanwhile, there are also some DNA enzymes in the body [71, 72], which degrade NETs and release their DNA and the protein components, promoting coagulation.

### 4.2. NETs Participate in Thrombosis

As mentioned above, NETs activate coagulation, indicating that they participate in thrombosis. Many histopathological studies have shown that NETs are involved in arterial thrombosis and venous thrombosis. Fuchs et al. [5] found that plasma DNA levels were elevated after the induction of deep vein thrombosis (DVT) and that NETs stimulated DVTs in baboons. Subsequent studies confirmed that neutrophils are crucial for the process of thrombosis and blocking the formation of NETs prevents thrombus formation [43]. Host DNases can prevent vascular occlusion induced by thrombosis via targeting NETs [71]. In addition, one clinical study has reported that NETs are present in thrombi in patients with DVT and chronic thromboembolic pulmonary hypertension (CTEPH) and mainly exist in the organizing thrombus. Moreover, dsDNA, MPO, NE, and MPO-DNA complex plasma levels are significantly higher in patients with CTEPH than those in healthy volunteers [22]. NETs are also involved in coronary thrombosis; coronary thrombi contain more NETs than venous thrombi and in vitro clots [73]. Studies have found that thrombi formed by NETs cannot be fully degraded by tissue plasminogen activator (tPA) and can only be prevented by a combination of tPA and DNase I treatment [5]. Mangold et al. [73] further confirmed that DNase can assist tPA to accelerate the dissolution of coronary thrombi. In addition, Longstaff et al. [74] found that addition of histone–DNA complexes to fibrin resulted in thicker fibers accompanied by improved stability and rigidity. Varjú I et al. [75] further demonstrated that DNA, histones, and NETs exerted antifibrinolytic effects through altering the fibrin architecture in plasma clots, while NETs contribute to a decreased lytic susceptibility that can be overcome by DNase.

### 4.3. Effects of NETs on Endothelial Cells

The endothelium plays an important role in thrombosis, and many studies have demonstrated that NETs can induce endothelial dysfunction [18, 76, 77]. NETs can induce endothelial cells (ECs) to release adhesion factors and TF, further recruiting inflammatory cells and promoting thrombosis [77]. Matrix metalloproteinase-9 (MMP-9) in NETs induces apoptosis of ECs through activating matrix metalloproteinase-2 (MMP-2) [18]. Pieterse et al. [78] further found that ECs had a limited ability to internalize NETs, while excessive phagocytic capacity of ECs for NETs resulted in cytotoxicity. Saffarzadeh et al. [79] confirmed that NETs induced cytotoxicity in a concentration-dependent manner. However, Aldabbous et al. [22] found that low concentrations of NETs (0.3 *μ*g/ml) induce ECs to release inflammatory factors and promote angiogenesis via the TLR4/NF-*κ*B signaling pathway, while high concentrations of NETs induce EC death [79]. Interestingly, the cytotoxicity of NETs from different sources is different. Compared with spontaneously formed NETs, the cytotoxicity of LPS-induced NETs to EC is more severe [80].

**Figure 2:**
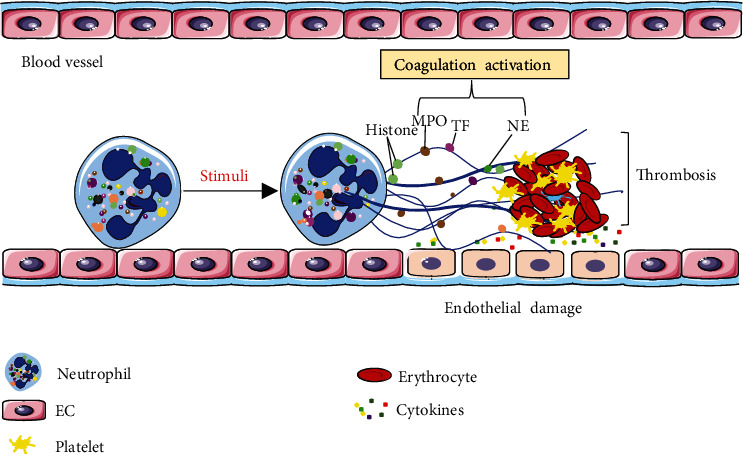
NETs participate in thrombosis. Under different stimuli, neutrophils release NETs. The proteins binding to NETs can activate the coagulation system and damage ECs. NETs also provide a scaffold for plasma protein and cell binding. Subsequently, NETs promote thrombosis formation.

The above research suggests that NETs are involved in the formation and development of thrombosis (Figure 2), and the level of NETs in peripheral blood is significantly correlated with thrombotic disease. Several studies have also found that DNase in addition to tPA can accelerate lysis of thrombi [5, 73], suggesting that NET-induced thrombi are different from those formed by fibrin coagulation. These studies point to the possible reasons for the poor clinical effects of conventional thrombolytic therapy and provide a new target for thrombolytic therapy: NETs.

## 5. NETs and Atherosclerosis

### 5.1. NETs Involved in the Formation and Development of Atherosclerosis

Atherosclerosis is a chronic inflammatory process. Inflammation plays an important role in the occurrence and development of atherosclerotic plaques [81]. Many studies have shown that neutrophils play an important role in the development of atherosclerosis [82–85]. In 2015, Warnatsch et al. [8] first reported that NETs are involved in the formation and development of advanced atherosclerotic plaques in vivo and that DNase I treatment can significantly inhibit the development of plaques. High cholesterol, hyperlipidemia, and hypertension are three known risk factors for atherosclerosis. Cholesterol can induce neutrophils to secrete NETs, which further promote macrophages to secrete inflammatory factors [8]. In addition, the levels of NETs in plasma and plaques are significantly increased under a high fat diet, and blocking NET formation by deleting the *PAD4* gene inhibits the expression of inflammatory factors in the aortic region and decreases the plaque area in the aortic root [86]. In vitro, the PAD4 inhibitor Cl-amidine also reduces the area of atherosclerotic plaque and the inflammatory response of the aorta in a photochemical damage mouse model [87]. However, *PAD4* knockout did not affect the formation of fat streaks, plaque size, and the inflammatory response, and PAD4 depletion or DNase treatment reduced arterial intimal damage and thrombosis formation in LDL gene knockout mice [88].

### 5.2. Clinical Research on NETs and Atherosclerosis

Many clinical studies have shown that plaque erosion can also cause acute coronary syndrome (ACS) due to vascular obstruction [89–92]. Histopathological studies have revealed that NETs are present on the luminal surface of eroded plaques, and NETs can promote ECs apoptosis and detachment in vitro, suggesting that NETs may be involved in plaque erosion [88, 93]. A recent autopsy study found that the level of NETs in plaques with thrombotic complication was significantly higher compared with these intact atherosclerotic plaques, while the level of macrophage extracellular traps (METs) was higher in intact plaques [94]. In addition, NETs are also involved in coronary artery thrombosis, and neutrophils isolated from blood samples obtained by infarct-related coronary arteries (IRA) have a stronger ability to form NETs compared with those isolated from blood samples obtained by non-infarct-related coronary arteries (non-IRA) [62]. Compared with the late coronary artery thrombi, the level of NETs is higher in early fresh thrombi, while METs are mainly present in late coronary artery thrombi [94]. This is opposite to the distribution of NETs in thrombi in CTEPH patients [95], suggesting that NETs may play different roles in arterial thrombosis and venous thrombosis. A later clinical study has found that plasma dsDNA, nucleosomes, and citH4 are increased in patients with severe coronary atherosclerosis [24]. Further, the plasma dsDNA levels are significantly higher in coronary artery blood than those in peripheral artery blood in myocardial infarction [25]. However, there is still relatively little research on the relationship between NETs and atherosclerotic cardiovascular diseases, and the detailed mechanism of the involvement of NETs in atherosclerosis is still unclear. Further work in this area is thus still required. Studies of the association between NETs and atherosclerosis are summarized in Table 3.

## 6. NETs and Autoimmune Diseases

Autoimmune diseases are mainly caused by immune system disorders in which immune cells cannot distinguish self-antigens from foreign ones and the body produces autoantibodies or cytotoxic T cells against its own tissues and organs [96]. Currently, many studies have demonstrated that the components of NETs are a source of autoantigens and induce immune cells to produce autoantibodies and play an important role in autoimmune diseases, such as vasculitis, systemic lupus erythematosus, and rheumatoid arthritis [97, 98]. Eighteen associations between NETs and autoimmune diseases were identified by PubMed search (Table 4).

### 6.1. NETs and Antineutrophil Cytoplasmic Autoantibody-Associated Vasculitis

Antineutrophil cytoplasmic antibody- (ANCA-) associated vasculitis (AAV) is a group of diseases, characterized by the destruction and inflammation of small vessels. Anti-MPO and proteinase 3 (PR3) antibodies are the two common ANCAs [99]. Kessenbrock et al. [100] first reported that NETs are present in the kidneys of AAV patients. ANCA-IgG can further induce the formation of NETs, which in turn promotes plasmacytoid dendritic cells (pDCs) to secrete interferon *α* (IFN-*α*). Excessive IFN-*α* can induce autoimmune phenomena, thus precipitating autoimmune diseases [101, 102]. In addition, myeloid DCs uploaded with NETs components can induce ANCA and autoimmunity when injected into naive mice, which can be prevented by treatment with DNase I [103]. Blocking NET formation by PAD inhibitors can suppress MPO-ANCA production in MPO-ANCA productizing mouse models, indicating that excessive formation of NETs may be correlated with MPO-ANCA production in vivo [35]. In addition, lysosomal membrane protein-2, a new type of ANCA autoantibody, is also involved in ANCA-induced NET formation in human neutrophils and is present in kidneys of AAV patients [104–108].

### 6.2. NETs and Systemic Lupus Erythematosus

Systemic lupus erythematosus (SLE) is a chronic disease that causes inflammation in connective tissues and is characterized by the production of antibodies to autologous dsDNA [109, 110]. NETs contain abundant dsDNA, which can induce the production of anti-dsDNA antibodies. In addition, antiribonucleoprotein antibodies in SLE patients can also induce neutrophils in SLE patients to secrete NETs, further activating pDCs to release proinflammatory factors, such as IFN-*α* and IL-6 [111]. Guo et al. [112] further confirmed that neutrophils derived from SLE patients were more likely to form NETs when stimulated by external stimuli, and SLE patients have a higher levels of IFN-*α*. In addition, neutrophil intracellular MMP-9 is externalized during NET formation, and MMP-9 induces endothelial dysfunction by activating MMP-2, which participates in the SLE disease process [18]. In vivo, there also exist anti-DNase in the plasma of SLE patients, which protects NETs from degradation [113]. Animal experiments revealed that neutrophils in an SLE mouse model have a significantly stronger ability to release NETs than normal mice [114]. However, blocking NET formation by knocking out of *NOX2* gene does not improve SLE symptoms in mouse. *NOX2* deletion not only inhibits the formation of NETs but also activates other inflammatory responses in the body, which may aggravate SLE symptoms [115]. Therefore, further research is required to determine how the formation of NETs in SLE can be blocked.

### 6.3. NETs and Rheumatoid Arthritis

Rheumatoid arthritis (RA) is a long-term chronic inflammatory process and mainly affects joints. Citrullinated proteins are the most important target antigens in the pathogenesis of RA [116, 117]. Spengler et al. [118] reported that neutrophils from RA patients released PADs, which mediate histone citrullination and play an important role in NET formation [37, 40]. In addition, neutrophils from RA patients display a significantly enhanced capacity to form NETs, and RA autoantibodies and inflammatory cytokines can induce NETs formation [119]. NETs also stimulate the externalization of citrullinated autoantigens and augment the inflammatory response in RA [119]. Blocking the formation of NETs by deletion of the *PAD4* gene reduces the severity of arthritis induced by recombinant human glucose-6-phosphate isomerase [120]. However, Rohrbach et al. [121] reported that knockout of *PAD4* gene did not affect the severity of arthritis in the K/BxN mice, suggesting that PAD4 and NETs might not play a role in autoantibody-mediated arthritis in a K/BxN mice model. In addition, NETs also carry the nuclear chromatin protein DEK, which participates in the development of arthritis [122]. Many clinical studies have demonstrated that neutrophils from RA patients exhibit enhanced NET formation; the serum markers of NETs (nucleosomes, NE, MPO, and MPO-DNA complex) are elevated, and that NETs-derived products may have potential clinical utility for the diagnosis of RA [123–125].

## 7. NETs and Sepsis

Sepsis is a life-threatening bloodstream infection that is accompanied by systemic inflammation and can cause multiple organ dysfunction. Neutrophils, the most abundant inflammatory cells in peripheral blood, are rapidly recruited and infiltrate into organs during the process of sepsis [126]. A large number of studies have shown that pathogenic microorganisms or their secreted products can stimulate neutrophils to form NETs [16, 127, 128]. NETs can capture pathogens, degrade bacterial toxic factors, and kill bacteria via their attached protein granzymes [3], which play an important role in the cure of sepsis. However, in recent years, many studies have found that NETs further aggravate tissue damage in sepsis [129–131]. NETs can induce macrophage pyroptosis and release inflammatory factors that augment inflammation in sepsis [132]. McDonald et al. [133] observed high levels of NETs in the liver vasculature of endotoxemic mice. Inhibition of the formation of NETs significantly inhibits intravascular coagulation, improves the reperfusion of blood vessels, and attenuates the end-organ damage in bacterial sepsis [133]. Biron et al. [134] further demonstrated that inhibition of NET formation by PAD4 inhibitors could significantly improve survival in a murine sepsis model. However, McDonald et al. [135] reported that NETs play an important role in protection against bacterial dissemination during sepsis and blocking NET formation significantly inhibited the capture of circulating bacteria. In addition, depletion of NETs by DNase also impairs the early immune response and aggravates the pathology following polymicrobial sepsis [136]. However, combining antibiotics and DNase in the treatment of sepsis can effectively reduce the damage of bacterial transmission to other organs (antibiotics) and can also inhibit organ damage by NETs through depletion of NETs (DNase) [137]. These studies suggested that blocking or depletion of NETs in the routine treatment of sepsis is beneficial for improving the outcome of sepsis. Studies of the association between NETs and sepsis are summarized in Table 5.

## 8. Conclusion

In recent years, research on NETs has attracted much attention in the field of immunology. The progress made give us a greater understanding of their role in immune diseases. Here, our focus has mainly been on three aspects: methods for detecting NETs, molecular mechanisms of NET formation, and the correlation between NET formation and disease. In this review, we summarized the detection targets of NETs in the blood, cells, and tissues based on previous reports and our own work. Plasma dsDNA, nucleosomes, MPO-DNA complexes (MPO or NE), and citH3 (citH4) are in vivo markers of NETs. In cells or tissues, the detection of NETs is a combination of dsDNA, citH3, and MPO (NE) by immunofluorescence. However, our suggestions are only for researchers' reference owing to lack of a unified NETs detection standard, and further work is required to establish a guideline for NETs detection [28]. We also discussed the regulatory mechanism of NETs formation. Independent pathways have been identified resulting in a morphologically similar outcome. NADPH and PAD4 play important roles in the regulation of NET formation, and a plethora of upstream and downstream signaling molecules have been studied. At last, we discussed the role of NETs-related diseases. Depletion or blocking NETs formation can reduce organ dysfunction and improve survival. However, NETs also serve protective effects by containing invading pathogens. Therefore, targeting of NETs to reduce organ damage should be cautiously adopted to the specific disease of interest.

## Figures and Tables

**Table 1:** The detection targets and methods of NETs in cells, tissues, and blood.

Sample type	Detection target	Detection method	Advantage	Limitations
Cell or tissue	dsDNA	SYTOX Green nucleic acid dye	Simple and direct	Less specific
	DNA, citH3, and MPO (or NE)	Immunofluorescence	Specific and widely accepted	Qualitative rather than quantitative
Blood	dsDNA	SYTOX Green nucleic acid dye	Simple and easy	Less specific
	dsDNA, nucleosomes, MPO-DNA complexes (MPO or NE), and citH3 (citH4)	ELISA	Specific and widely accepted	—

**Table 2:** Comparison of the three types of NET formation.

Type	Main process	Neutrophil survival status	Time
Vesicle-mediated	Nuclear envelope breakdown and vesicle formation	Alive	5–60 min
Cell lytic	Histone citrullination and cell membrane rupture	Dead	180–240 min
Mitochondrial DNA	Unclear	Alive	>35 min

**Table 3:** List of studies showing association between NETs and atherosclerosis.

Authors, reference	Study design	Main study findings
Warnatsch et al. [8]	Using a mouse model	Cholesterol crystals triggered neutrophils to release NETs. NETs primed macrophages for cytokine production in atherosclerosis and blocking NETs formation significantly attenuated the plaque progression.
Liu et al. [86]	Using a mouse model	NETs are present in atherosclerotic lesions and are associated with the development of atherosclerosis. Specific deletion of PAD4 in the myeloid lineage diminished NET formation and significantly reduced atherosclerosis burden.
Knight et al. [87]	Using a mouse model	Pharmacological interventions that block NET formation via targeting PAD4 can reduce atherosclerosis burden and arterial thrombosis
Franck et al. [88]	Using a mouse model	NETs do not influence chronic experimental atherogenesis, but participate causally in acute thrombotic complications of intimal lesions that recapitulate features of superficial erosion.
Quillard et al. [93]	Analyzing 56 specimens of human carotid plaques.	NETs are present in human atherosclerotic plaques and associate with the number of luminal apoptotic ECs.
Pertiwi et al. [94]	Analyzing 12 thrombosed plaques obtained at autopsy from patients with acute MI.	NETs dominate numerically in early thrombosis and macrophage traps in late (organizing) thrombosis.
Stakos et al. [62]	Cell experiments (neutrophils obtained from patients with STEMI)	Neutrophils isolated from blood samples obtained by IRA have a higher ability to form NETs compared with those isolated from blood samples obtained by non-IRA.

NET: neutrophil extracellular traps; PAD4: peptidylarginine deiminase 4; ECs: endothelial cells; MI: myocardial infarction; STEMI: ST-elevation myocardial infarction; IRA: infarct-related coronary arteries.

**Table 4:** List of studies showing association between NETs and autoimmune diseases.

Authors, reference	Study design	Main study findings
Kessenbrock et al. [100]	Cell experiments (neutrophils isolated from human peripheral blood) and analyzing 15 kidney needle biopsies from SVV patients with glomerulonephritis.	NETs are released by ANCA-stimulated neutrophils and contain the targeted autoantigens PR3 and MPO. NETs were prominent in specimens with strong neutrophil infiltration.
Sangaletti et al. [103]	Using a mouse model	Myeloid DCs uploaded with and activated by NET components induce ANCA and autoimmunity. NET intermingling with myeloid DC positive for neutrophil MPO in MPO-ANCA-associated microscopic polyangiitis.
Kusunoki et al. [35]	Using a mouse model	PAD inhibitor suppresses NETs formation and MPO-ANCA production in mouse models with MPO-ANCA production.
Tang et al. [104] [97]	Cell experiments (neutrophils isolated from human peripheral blood) and analyzing 6 kidney needle biopsies from AVV patients.	Enhanced NET formation, which contains LAMP-2, was observed in kidney biopsies and neutrophils from AAV patients. Anti-LAMP-2 antibody can further promote NETs formation.
Garcia-Romo et al. [111]	Analyzing the human neutrophils.	Mature SLE neutrophils are primed in vivo by type I IFN and die upon exposure to SLE-derived antiribonucleoprotein antibodies, releasing NETs. SLE NETs facilitate the uptake and recognition of mammalian DNA by pDCs and activate pDCs to produce high levels of IFN-*α*.
Guo et al. [112]	Analyzing the human neutrophils.	Neutrophils derived from SLE patients with decreased RIPK1 expression are more likely to form NETs, and RIPK1 inhibitor can greatly increase NETs formation.
Carmona-Rivera et al. [18]	Cell experiments.	MMP-9 is externalized during NET formation, and MMP-9 induces endothelial dysfunction by activating MMP-2. Inhibition of MMP-2 activation can restore endothelium-dependent function and decreased NET-induced vascular cytotoxicity.
Hakkim et al. [113]	Analysis of sera from 61 unrelated patients with SLE, 54 healthy controls, 30 RA patients, and 4 patients with IgA nephropathy.	A subset of SLE patients' sera DNase I inhibitors or anti-NET antibodies prevented DNase1 access to NETs.
Knight et al. [114]	Using a mouse model	Neutrophils in SLE mouse model have a significantly higher ability to release NETs compared with controls. PAD inhibition can reduce NET formation, protecting against lupus-related damage to the vasculature, kidneys, and skin.
Campbell et al. [115] [107]	Using a mouse model	NETs does not contribute to SLE in Nox2-deficient lupus-prone mice.
Spengler et al. [118]	Analysis of synovial fluid from patients with RA, patients with osteoarthritis, and patients with psoriatic arthritis.	Extracellular DNA levels were significantly higher in RA patient than in OA patients, and correlated with neutrophil concentrations and PAD activity in RA. PAD2 and PAD4 were attached to NETs and also freely diffused in the supernatant.
Khandpur et al. [119]	Experiments using neutrophils, sera, and synovial fluid obtained from RA patients, healthy controls, and patients with osteoarthritis.	Neutrophils from RA patients display a significantly enhanced capacity to form NETs, and NETs are a source of citrullinated autoantigens and stimulate inflammatory responses in RA.
Seri et al. [120]	Using a mouse model	Deletion of the PAD4 gene reduces the severity of arthritis induced by recombinant human glucose-6-phosphate isomerase.
Rohrbach et al. [121]	Using a mouse model	PAD4 deficiency did not affect the severity of arthritis in the K/BxN murine.
Mor-Vaknin et al. [122]	Using a mouse model	DEK is detected in spontaneously forming NETs from JIA patient synovial neutrophils, and DEK-targeted aptamers significantly reduces joint inflammation in vivo and greatly impairs the ability of neutrophils to form NETs.
Perez-Sanchez et al. [123]	Experiments using neutrophils and plasma obtained from RA patients and healthy controls.	NETs was found increased in RA patients. Inhibition of NETs extrusion can further abridge the endothelial dysfunction and the activation of immune cells, thus influencing the global activity of the vascular system.
Sur Chowdhury et al. [124]	Human study.	Neutrophils from RA cases exhibited increased spontaneous NET formation, and NETs-derived in RA serum products demonstrated diagnostic potential.
Wang et al. [125]	Analysis of serum from 74 RA patients and 50 healthy controls.	RA patients exhibited significantly higher levels of MPO-DNA complexes, and these levels were associated with increased neutrophil counts and positivity for rheumatoid factor and anticitrullinated protein/peptide antibodies.

NET: neutrophil extracellular traps; ANCA: antineutrophil cytoplasmic antibody; SVV: small-vessel vasculitis; PR3: proteinase 3; MPO: myeloperoxidase; DCs: dendritic cells; PAD: peptidylarginine deiminase; LAMP-2: lysosomal-associated membrane protein-2; pDCs: plasmacytoid dendritic cells; IFN: interferon; AAV: ANCA-associated vasculitis; RIPK1: receptor interacting serine/threonine kinase 1; SLE: systemic lupus erythematosus RA: rheumatoid arthritis; MMP: matrix metalloproteinase; OA: osteoarthritis; JIA: juvenile idiopathic arthritis.

**Table 5:** Studies showing an association between NETs and sepsis.

Authors, reference	Study design	Main study findings
Brinkmann et al. [3]	Cell experiments.	Upon activation, neutrophils release NETs that bind Gram-positive and Gram-negative bacteria. NETs further degrade virulence factors and kill bacteria.
Yang et al. [129]	Analyzing neutrophils, platelets and plasma obtained from sepsis patients, nonsepsis patients and healthy controls.	Neutrophils from septic patients had significantly enhanced NETs releasing. NETs further promote hypercoagulability in patients with sepsis.
Lefrancais et al. [130]	Human and mouse study.	They detected NETs in abundance in mouse models of severe bacterial pneumonia/acute lung injury and in human subjects with acute respiratory distress syndrome from pneumonia or sepsis. Increased plasma NETs were associated with ARDS severity and mortality in humans.
Tanaka et al. [131]	Using a mouse model	In septic mice, NETs were significantly increased in postcapillary venules of the cecum and hepatic sinusoids with increased leukocyte-endothelial interactions. NETs were also observed in both alveolar space and pulmonary capillaries of the lung.
Chen et al. [132]	Using a mouse model.	NETs induce M*ϕ* pyroptosis in sepsis. M*ϕ* pyroptosis further augments inflammatory responses following sepsis.
McDonald et al. [133]	Using a mouse model.	NETs were critical for the development of sepsis-induced intravascular coagulation in mice. Inhibition of NET-induced coagulation can markedly improve microvascular perfusion and attenuate the end-organ damage in septic mice.
Biron et al. [134]	Using a mouse model.	Cl-Amidine (PAD4 inhibitor) treatment prior to cecal ligation and puncture improves overall survival in sepsis.
McDonald et al. [135]	Using a mouse model.	NET release increases bacterial trapping, and blocking NET formation reduces the capture of circulating bacteria during sepsis, resulting in increased dissemination to distant organs.
Meng et al. [136]	Using a mouse model.	They found that depletion of NETs by rhDNase administration can impede the early immune response and aggravates the pathology that follows polymicrobial sepsis.
Czaikoski et al. [137]	Using a mouse model.	Degradation of NETs by rhDNase treatment did not prevent organ damage during polymicrobial sepsis, while rhDNase plus antibiotics attenuated sepsis-induced organ damage and improved the survival rate.

NET: neutrophil extracellular traps; ARDS: acute respiratory distress syndrome; M*ϕ*: macrophage; PAD4: peptidylarginine deiminase 4; rhDNase: recombinant human deoxyribonuclease.

## References

[1] Hellebrekers P., Vrisekoop N., Koenderman L. (2018). Neutrophil phenotypes in health and disease. *European Journal of Clinical Investigation*.

[2] Kobayashi S. D., Malachowa N., DeLeo F. R. (2018). Neutrophils and bacterial immune evasion. *Journal of Innate Immunity*.

[3] Brinkmann V., Reichard U., Goosmann C. (2004). Neutrophil extracellular traps kill bacteria. *Science*.

[4] Li R. H. L., Tablin F. (2018). A comparative review of neutrophil extracellular traps in sepsis. *Frontiers in Veterinary Science*.

[5] Fuchs T. A., Brill A., Duerschmied D. (2010). Extracellular DNA traps promote thrombosis. *Proceedings of the National Academy of Sciences of the United States of America*.

[6] Engelmann B., Massberg S. (2013). Thrombosis as an intravascular effector of innate immunity. *Nature Reviews. Immunology*.

[7] Alhamdi Y., Toh C. H. (2017). Recent advances in pathophysiology of disseminated intravascular coagulation: The role of circulating histones and neutrophil extracellular traps. *F1000Res*.

[8] Warnatsch A., Ioannou M., Wang Q., Papayannopoulos V. (2015). Inflammation. Neutrophil extracellular traps license macrophages for cytokine production in atherosclerosis. *Science*.

[9] Lee K. H., Kronbichler A., Park D. D. Y. (2017). Neutrophil extracellular traps (nets) in autoimmune diseases: a comprehensive review. *Autoimmunity Reviews*.

[10] Berezin A. (2019). Neutrophil extracellular traps: the core player in vascular complications of diabetes mellitus. *Diabetes and Metabolic Syndrome: Clinical Research and Reviews*.

[11] Wong S. L., Demers M., Martinod K. (2015). Diabetes primes neutrophils to undergo netosis, which impairs wound healing. *Nature Medicine*.

[12] Pietronigro E. C., Della Bianca V., Zenaro E., Constantin G. (2017). Netosis in Alzheimer's disease. *Front Immunol*.

[13] Thalin C., Hisada Y., Lundstrom S., Mackman N., Wallen H. (2019). Neutrophil extracellular traps: villains and targets in arterial, venous, and cancer-associated thrombosis. *Arteriosclerosis, Thrombosis, and Vascular Biology*.

[14] Erpenbeck L., Schon M. P. (2017). Neutrophil extracellular traps: protagonists of cancer progression?. *Oncogene*.

[15] Leppkes M., Maueröder C., Hirth S. (2016). Externalized decondensed neutrophil chromatin occludes pancreatic ducts and drives pancreatitis. *Nature communications*.

[16] Dabrowska D., Jablonska E., Garley M., Ratajczak-Wrona W., Iwaniuk A. (2016). New aspects of the biology of neutrophil extracellular traps. *Scandinavian Journal of Immunology*.

[17] Urban C. F., Ermert D., Schmid M. (2009). Neutrophil extracellular traps contain calprotectin, a cytosolic protein complex involved in host defense against candida albicans. *PLoS Pathogens*.

[18] Carmona-Rivera C., Zhao W., Yalavarthi S., Kaplan M. J. (2015). Neutrophil extracellular traps induce endothelial dysfunction in systemic lupus erythematosus through the activation of matrix metalloproteinase-2. *Annals of the Rheumatic Diseases*.

[19] Albrengues J., Shields M. A., Ng D. (2018). Neutrophil extracellular traps produced during inflammation awaken dormant cancer cells in mice. *Science*.

[20] Kambas K., Mitroulis I., Ritis K. (2012). The emerging role of neutrophils in thrombosis-the journey of tf through nets. *Front Immunol*.

[21] Li T., Peng R., Wang F. (2020). Lysophosphatidic acid promotes thrombus stability by inducing rapid formation of neutrophil extracellular traps: A new mechanism of thrombosis. *Journal of Thrombosis and Haemostasis*.

[22] Aldabbous L., Abdul-Salam V., McKinnon T. (2016). Neutrophil extracellular traps promote angiogenesis. *Arteriosclerosis, Thrombosis, and Vascular Biology*.

[23] Marcos V., Zhou Z., Yildirim A. Ö. (2010). CXCR2 mediates NADPH oxidase-independent neutrophil extracellular trap formation in cystic fibrosis airway inflammation. *Nature Medicine*.

[24] Borissoff J. I., Joosen I. A., Versteylen M. O. (2013). Elevated levels of circulating DNA and chromatin are independently associated with severe coronary atherosclerosis and a prothrombotic state. *Arteriosclerosis, Thrombosis, and Vascular Biology*.

[25] Wang X., Yang D., Liu J., Fan X., Ma A., Liu P. (2018). Prognostic value of culprit artery double-stranded DNA in st-segment elevated myocardial infarction. *Scientific Reports*.

[26] Zhang S., Lu X., Shu X. (2014). Elevated plasma cfDNA may be associated with active lupus nephritis and partially attributed to abnormal regulation of neutrophil extracellular traps (nets) in patients with systemic lupus erythematosus. *Internal Medicine*.

[27] Phillipson M., Kubes P. (2011). The neutrophil in vascular inflammation. *Nature Medicine*.

[28] Boeltz S., Amini P., Anders H. J. (2019). To NET or not to NET:current opinions and state of the science regarding the formation of neutrophil extracellular traps. *Cell Death and Differentiation*.

[29] Pilsczek F. H., Salina D., Poon K. K. H. (2010). A novel mechanism of rapid nuclear neutrophil extracellular trap formation in response toStaphylococcus aureus. *Journal of Immunology*.

[30] Papayannopoulos V., Metzler K. D., Hakkim A., Zychlinsky A. (2010). Neutrophil elastase and myeloperoxidase regulate the formation of neutrophil extracellular traps. *The Journal of Cell Biology*.

[31] Yousefi S., Mihalache C., Kozlowski E., Schmid I., Simon H. U. (2009). Viable neutrophils release mitochondrial DNA to form neutrophil extracellular traps. *Cell Death and Differentiation*.

[32] Keshari R. S., Jyoti A., Kumar S. (2012). Neutrophil extracellular traps contain mitochondrial as well as nuclear DNA and exhibit inflammatory potential. *Cytometry. Part A*.

[33] Lood C., Blanco L. P., Purmalek M. M. (2016). Neutrophil extracellular traps enriched in oxidized mitochondrial DNA are interferogenic and contribute to lupus-like disease. *Nature Medicine*.

[34] Rohrbach A. S., Slade D. J., Thompson P. R., Mowen K. A. (2012). Activation of pad4 in net formation. *Front Immunol*.

[35] Kusunoki Y., Nakazawa D., Shida H. (2016). Peptidylarginine deiminase inhibitor suppresses neutrophil extracellular trap formation and mpo-anca production. *Front Immunol*.

[36] Li P., Li M., Lindberg M. R., Kennett M. J., Xiong N., Wang Y. (2010). PAD4 is essential for antibacterial innate immunity mediated by neutrophil extracellular traps. *The Journal of Experimental Medicine*.

[37] Wong S. L., Wagner D. D. (2018). Peptidylarginine deiminase 4: a nuclear button triggering neutrophil extracellular traps in inflammatory diseases and aging. *Frontiers in Veterinary Science*.

[38] Nakashima K., Hagiwara T., Ishigami A. (1999). Molecular characterization of peptidylarginine deiminase in hl-60 cells induced by retinoic acid and 1alpha,25-dihydroxyvitamin d(3). *The Journal of Biological Chemistry*.

[39] Nakashima K., Hagiwara T., Yamada M. (2002). Nuclear localization of peptidylarginine deiminase v and histone deimination in granulocytes. *The Journal of Biological Chemistry*.

[40] Wang Y., Li M., Stadler S. (2009). Histone hypercitrullination mediates chromatin decondensation and neutrophil extracellular trap formation. *The Journal of Cell Biology*.

[41] Lewis H. D., Liddle J., Coote J. E. (2015). Inhibition of pad4 activity is sufficient to disrupt mouse and human net formation. *Nature Chemical Biology*.

[42] Saha P., Yeoh B. S., Xiao X. (2019). PAD4-dependent NETs generation are indispensable for intestinal clearance of _Citrobacter rodentium_. *Mucosal immunology*.

[43] Martinod K., Demers M., Fuchs T. A. (2013). Neutrophil histone modification by peptidylarginine deiminase 4 is critical for deep vein thrombosis in mice. *Proceedings of the National Academy of Sciences of the United States of America*.

[44] Neeli I., Khan S. N., Radic M. (2008). Histone deimination as a response to inflammatory stimuli in neutrophils. *Journal of Immunology*.

[45] Tilvawala R., Nguyen S. H., Maurais A. J. (2018). The rheumatoid arthritis-associated citrullinome. *Cell chemical biology*.

[46] Gößwein S., Lindemann A., Mahajan A. (2019). Citrullination licenses calpain to decondense nuclei in neutrophil extracellular trap formation. *Front Immunol*.

[47] Douda D. N., Khan M. A., Grasemann H., Palaniyar N. (2015). SK3 channel and mitochondrial ROS mediate NADPH oxidase-independent netosis induced by calcium influx. *Proceedings of the National Academy of Sciences of the United States of America*.

[48] Parker H., Dragunow M., Hampton M. B., Kettle A. J., Winterbourn C. C. (2012). Requirements for NADPH oxidase and myeloperoxidase in neutrophil extracellular trap formation differ depending on the stimulus. *Journal of Leukocyte Biology*.

[49] Kenny E. F., Herzig A., Krüger R. (2017). Diverse stimuli engage different neutrophil extracellular trap pathways. *Elife*.

[50] Neeli I., Dwivedi N., Khan S., Radic M. (2009). Regulation of extracellular chromatin release from neutrophils. *Journal of Innate Immunity*.

[51] Awasthi D., Nagarkoti S., Kumar A. (2016). Oxidized ldl induced extracellular trap formation in human neutrophils via tlr-pkc-irak-mapk and NADPH-oxidase activation. *Free Radic Biol Med*.

[52] McInturff A. M., Cody M. J., Elliott E. A. (2012). Mammalian target of rapamycin regulates neutrophil extracellular trap formation via induction of hypoxia-inducible factor 1 *α*. *Blood*.

[53] Itakura A., McCarty O. J. T. (2013). Pivotal role for the mtor pathway in the formation of neutrophil extracellular traps via regulation of autophagy. *American Journal of Physiology. Cell Physiology*.

[54] Hakkim A., Fuchs T. A., Martinez N. E. (2011). Activation of the raf-mek-erk pathway is required for neutrophil extracellular trap formation. *Nature Chemical Biology*.

[55] Neeli I., Radic M. (2013). Opposition between pkc isoforms regulates histone deimination and neutrophil extracellular chromatin release. *Front Immunol*.

[56] DeSouza-Vieira T., Guimarães-Costa A., Rochael N. C. (2016). Neutrophil extracellular traps release induced byLeishmania: role of PI3K*γ*, erk, PI3K*σ*, pkc, and [ca2+]. *Journal of Leukocyte Biology*.

[57] Gavillet M., Martinod K., Renella R., Wagner D. D., Williams D. A. (2018). A key role for rac and pak signaling in neutrophil extracellular traps (nets) formation defines a new potential therapeutic target. *American Journal of Hematology*.

[58] Liu L., Mao Y., Xu B. (2019). Induction of neutrophil extracellular traps during tissue injury: involvement of sting and toll-like receptor 9 pathways. *Cell Proliferation*.

[59] Behnen M., Leschczyk C., Möller S. (2014). Immobilized immune complexes induce neutrophil extracellular trap release by human neutrophil granulocytes via Fc*γ*RIIIB and mac-1. *Journal of Immunology*.

[60] Massberg S., Grahl L., von Bruehl M. L. (2010). Reciprocal coupling of coagulation and innate immunity via neutrophil serine proteases. *Nature Medicine*.

[61] Glaser C. B., Morser J., Clarke J. H. (1992). Oxidation of a specific methionine in thrombomodulin by activated neutrophil products blocks cofactor activity. A potential rapid mechanism for modulation of coagulation. *The Journal of Clinical Investigation*.

[62] Stakos D. A., Kambas K., Konstantinidis T. (2015). Expression of functional tissue factor by neutrophil extracellular traps in culprit artery of acute myocardial infarction. *European Heart Journal*.

[63] Darbousset R., Thomas G. M., Mezouar S. (2012). Tissue factor-positive neutrophils bind to injured endothelial wall and initiate thrombus formation. *Blood*.

[64] Kambas K., Chrysanthopoulou A., Vassilopoulos D. (2014). Tissue factor expression in neutrophil extracellular traps and neutrophil derived microparticles in antineutrophil cytoplasmic antibody associated vasculitis may promote thromboinflammation and the thrombophilic state associated with the disease. *Annals of the Rheumatic Diseases*.

[65] Grover S. P., Mackman N. (2018). Tissue factor. *Arteriosclerosis, Thrombosis, and Vascular Biology*.

[66] von Brühl M.-L., Stark K., Steinhart A. (2012). Monocytes, neutrophils, and platelets cooperate to initiate and propagate venous thrombosis in mice in vivo. *The Journal of Experimental Medicine*.

[67] Kambas K., Mitroulis I., Apostolidou E. (2012). Autophagy mediates the delivery of thrombogenic tissue factor to neutrophil extracellular traps in human sepsis. *PLoS One*.

[68] Gould T. J., Vu T. T., Swystun L. L. (2014). Neutrophil extracellular traps promote thrombin generation through platelet-dependent and platelet-independent mechanisms. *Arteriosclerosis, Thrombosis, and Vascular Biology*.

[69] Noubouossie D. F., Whelihan M. F., Yu Y. B. (2017). In vitro activation of coagulation by human neutrophil DNA and histone proteins but not neutrophil extracellular traps. *Blood*.

[70] Clark S. R., Ma A. C., Tavener S. A. (2007). Platelet tlr4 activates neutrophil extracellular traps to ensnare bacteria in septic blood. *Nature Medicine*.

[71] Jiménez-Alcázar M., Rangaswamy C., Panda R. (2017). Host DNases prevent vascular occlusion by neutrophil extracellular traps. *Science*.

[72] Jiménez-Alcázar M., Napirei M., Panda R. (2015). Impaired DNase1-mediated degradation of neutrophil extracellular traps is associated with acute thrombotic microangiopathies. *Journal of Thrombosis and Haemostasis*.

[73] Mangold A., Alias S., Scherz T. (2015). Coronary neutrophil extracellular trap burden and deoxyribonuclease activity in st-elevation acute coronary syndrome are predictors of st-segment resolution and infarct size. *Circulation Research*.

[74] Longstaff C., Varjú I., Sótonyi P. (2013). Mechanical stability and fibrinolytic resistance of clots containing fibrin, DNA, and histones. *The Journal of Biological Chemistry*.

[75] Varjú I., Longstaff C., Szabó L. (2017). DNA, histones and neutrophil extracellular traps exert anti-fibrinolytic effects in a plasma environment. *Thrombosis and Haemostasis*.

[76] Rabinovitch M. (2016). NETs activate pulmonary arterial endothelial cells. *Arteriosclerosis, Thrombosis, and Vascular Biology*.

[77] Folco E. J., Mawson T. L., Vromman A. (2018). Neutrophil extracellular traps induce endothelial cell activation and tissue factor production through Interleukin-1*α* and cathepsin g. *Arteriosclerosis, Thrombosis, and Vascular Biology*.

[78] Pieterse E., Rother N., Garsen M. (2017). Neutrophil extracellular traps drive endothelial-to-mesenchymal transition. *Arteriosclerosis, Thrombosis, and Vascular Biology*.

[79] Saffarzadeh M., Juenemann C., Queisser M. A. (2012). Neutrophil extracellular traps directly induce epithelial and endothelial cell death: a predominant role of histones. *PLoS One*.

[80] Liang Y., Pan B., Alam H. B. (2018). Inhibition of peptidylarginine deiminase alleviates lps-induced pulmonary dysfunction and improves survival in a mouse model of lethal endotoxemia. *Eur J Pharmacol*.

[81] Wildgruber M., Swirski F. K., Zernecke A. (2013). Molecular imaging of inflammation in atherosclerosis. *Theranostics*.

[82] Carbone F., Nencioni A., Mach F., Vuilleumier N., Montecucco F. (2017). Pathophysiological role of neutrophils in acute myocardial infarction. *Thrombosis and Haemostasis*.

[83] Soehnlein O. (2012). Multiple roles for neutrophils in atherosclerosis. *Circulation Research*.

[84] Drechsler M., Megens R. T. A., van Zandvoort M., Weber C., Soehnlein O. (2010). Hyperlipidemia-triggered neutrophilia promotes early atherosclerosis. *Circulation*.

[85] Chistiakov D. A., Grechko A. V., Myasoedova V. A., Melnichenko A. A., Orekhov A. N. (2018). The role of monocytosis and neutrophilia in atherosclerosis. *Journal of Cellular and Molecular Medicine*.

[86] Liu Y., Carmona-Rivera C., Moore E. (2018). Myeloid-specific deletion of peptidylarginine deiminase 4 mitigates atherosclerosis. *Front Immunol*.

[87] Knight J. S., Luo W., O’Dell A. A. (2014). Peptidylarginine deiminase inhibition reduces vascular damage and modulates innate immune responses in murine models of atherosclerosis. *Circulation Research*.

[88] Franck G., Mawson T. L., Folco E. J. (2018). Roles of pad4 and netosis in experimental atherosclerosis and arterial injury: implications for superficial erosion. *Circulation Research*.

[89] Libby P., Pasterkamp G., Crea F., Jang I. K. (2019). Reassessing the mechanisms of acute coronary syndromes. *Circulation Research*.

[90] Sugiyama T., Yamamoto E., Bryniarski K. (2018). Nonculprit plaque characteristics in patients with acute coronary syndrome caused by plaque erosion vs plaque rupture: a 3-vessel optical coherence tomography study. *JAMA Cardiology*.

[91] Higuma T., Soeda T., Abe N. (2015). A Combined Optical Coherence Tomography and Intravascular Ultrasound Study on Plaque Rupture, Plaque Erosion, and Calcified Nodule in Patients With ST- Segment Elevation Myocardial Infarction: Incidence, Morphologic Characteristics, and Outcomes After Percutaneous Coronary Intervention. *JACC. Cardiovascular Interventions*.

[92] Hayashi T., Kiyoshima T., Matsuura M. (2005). Plaque erosion in the culprit lesion is prone to develop a smaller myocardial infarction size compared with plaque rupture. *American Heart Journal*.

[93] Quillard T., Araújo H. A., Franck G., Shvartz E., Sukhova G., Libby P. (2015). TLR2 and neutrophils potentiate endothelial stress, apoptosis and detachment: implications for superficial erosion. *European Heart Journal*.

[94] Pertiwi K. R., de Boer O. J., Mackaaij C. (2019). Extracellular traps derived from macrophages, mast cells, eosinophils and neutrophils are generated in a time-dependent manner during atherothrombosis. *The Journal of Pathology*.

[95] Savchenko A. S., Martinod K., Seidman M. A. (2014). Neutrophil extracellular traps form predominantly during the organizing stage of human venous thromboembolism development. *Journal of Thrombosis and Haemostasis*.

[96] Wu H., Liao J., Li Q., Yang M., Zhao M., Lu Q. (2018). Epigenetics as biomarkers in autoimmune diseases. *Clin Immunol*.

[97] Selmi C. (2017). Autoimmunity in 2016. *Clinical Reviews in Allergy & Immunology*.

[98] Soderberg D., Segelmark M. (2016). Neutrophil extracellular traps in anca-associated vasculitis. *Front Immunol*.

[99] Hsieh S. C., Yu H. S., Cheng S. H. (2006). Anti-myeloperoxidase antibodies enhance phagocytosis, il-8 production, and glucose uptake of polymorphonuclear neutrophils rather than anti-proteinase 3 antibodies leading to activation-induced cell death of the neutrophils. *Clinical Rheumatology*.

[100] Kessenbrock K., Krumbholz M., Schönermarck U. (2009). Netting neutrophils in autoimmune small-vessel vasculitis. *Nature Medicine*.

[101] Arai Y., Yamashita K., Kuriyama K. (2015). Plasmacytoid dendritic cell activation and IFN-alpha production are prominent features of murine autoimmune pancreatitis and human igg4-related autoimmune pancreatitis. *Journal of Immunology*.

[102] Chyuan I. T., Tzeng H. T., Chen J. Y. (2019). Signaling pathways of type I and type III interferons and targeted therapies in systemic lupus erythematosus. *Cells*.

[103] Sangaletti S., Tripodo C., Chiodoni C. (2012). Neutrophil extracellular traps mediate transfer of cytoplasmic neutrophil antigens to myeloid dendritic cells toward anca induction and associated autoimmunity. *Blood*.

[104] Tang S., Zhang Y., Yin S. W. (2015). Neutrophil extracellular trap formation is associated with autophagy-related signalling in anca-associated vasculitis. *Clinical and Experimental Immunology*.

[105] Kain R., Tadema H., McKinney E. F. (2012). High prevalence of autoantibodies to hlamp-2 in anti-neutrophil cytoplasmic antibody-associated vasculitis. *Journal of the American Society of Nephrology : JASN*.

[106] Bosch X., Mirapeix E. (2009). LAMP-2 illuminates pathogenesis of ANCA glomerulonephritis. *Nature Reviews. Nephrology*.

[107] Kain R., Exner M., Brandes R. (2008). Molecular mimicry in pauci-immune focal necrotizing glomerulonephritis. *Nature Medicine*.

[108] Roitsch S., Gößwein S., Neurath M. F., Leppkes M. (2018). Detection by flow cytometry of anti-neutrophil cytoplasmic antibodies in a novel approach based on neutrophil extracellular traps. *Autoimmunity*.

[109] Bai Y., Tong Y., Liu Y., Hu H. (2018). Self-dsDNA in the pathogenesis of systemic lupus erythematosus. *Clinical and Experimental Immunology*.

[110] Pravda J. (2019). Systemic lupus erythematosus: pathogenesis at the functional limit of redox homeostasis. *Oxid Med Cell Longev*.

[111] Garcia-Romo G. S., Caielli S., Vega B. (2011). Netting neutrophils are major inducers of type i ifn production in pediatric systemic lupus erythematosus. *Science translational medicine*.

[112] Guo R., Tu Y., Xie S. (2018). A role for receptor-interacting protein kinase-1 in neutrophil extracellular trap formation in patients with systemic lupus erythematosus: a preliminary study. *Cellular Physiology and Biochemistry*.

[113] Hakkim A., Furnrohr B. G., Amann K. (2010). Impairment of neutrophil extracellular trap degradation is associated with lupus nephritis. *Proceedings of the National Academy of Sciences of the United States of America*.

[114] Knight J. S., Subramanian V., O'Dell A. A. (2015). Peptidylarginine deiminase inhibition disrupts net formation and vprotects against kidney, skin and vascular disease in lupus-prone mrl/lpr mice. *Annals of the Rheumatic Diseases*.

[115] Campbell A. M., Kashgarian M., Shlomchik M. J. (2012). Nadph oxidase inhibits the pathogenesis of systemic lupus erythematosus. *Science translational medicine*.

[116] Farid S. S., Azizi G., Mirshafiey A. (2013). Anti-citrullinated protein antibodies and their clinical utility in rheumatoid arthritis. *International Journal of Rheumatic Diseases*.

[117] Savtekin G., Sehirli A. O. (2018). Rheumatoid arthritis in temporo-mandibular joint: a review. *Nigerian Journal of Clinical Practice*.

[118] Spengler J., Lugonja B., Jimmy Ytterberg A. (2015). Release of active peptidyl arginine deiminases by neutrophils can explain production of extracellular citrullinated autoantigens in rheumatoid arthritis synovial fluid. *Arthritis & Rhematology*.

[119] Khandpur R., Carmona-Rivera C., Vivekanandan-Giri A. (2013). Nets are a source of citrullinated autoantigens and stimulate inflammatory responses in rheumatoid arthritis. *Science translational medicine*.

[120] Seri Y., Shoda H., Suzuki A. (2015). _Peptidylarginine deiminase type 4_ deficiency reduced arthritis severity in a glucose-6-phosphate isomerase-induced arthritis model. *Sci Rep*.

[121] Rohrbach A. S., Hemmers S., Arandjelovic S., Corr M., Mowen K. A. (2012). PAD4 is not essential for disease in the k/bxn murine autoantibody-mediated model of arthritis. *Arthritis Research & Therapy*.

[122] Mor-Vaknin N., Saha A., Legendre M. (2017). Dek-targeting DNA aptamers as therapeutics for inflammatory arthritis. *Nature communications*.

[123] Perez-Sanchez C., Ruiz-Limon P., Aguirre M. A. (2017). Diagnostic potential of netosis-derived products for disease activity, atherosclerosis and therapeutic effectiveness in rheumatoid arthritis patients. *Journal of autoimmunity*.

[124] Sur Chowdhury C., Giaglis S., Walker U. A., Buser A., Hahn S., Hasler P. (2014). Enhanced neutrophil extracellular trap generation in rheumatoid arthritis: analysis of underlying signal transduction pathways and potential diagnostic utility. *Arthritis Research & Therapy*.

[125] Wang W., Peng W., Ning X. (2018). Increased levels of neutrophil extracellular trap remnants in the serum of patients with rheumatoid arthritis. *International Journal of Rheumatic Diseases*.

[126] Grommes J., Soehnlein O. (2011). Contribution of neutrophils to acute lung injury. *Molecular Medicine*.

[127] Gardiner E. E., Andrews R. K. (2012). Neutrophil extracellular traps (nets) and infection-related vascular dysfunction. *Blood Reviews*.

[128] Vorobjeva N. V., Pinegin B. V. (2014). Neutrophil extracellular traps: mechanisms of formation and role in health and disease. *Biochemistry (Mosc)*.

[129] Yang S., Qi H., Kan K. (2017). Neutrophil extracellular traps promote hypercoagulability in patients with sepsis. *Shock (Augusta, Ga.)*.

[130] Lefrancais E., Mallavia B., Zhuo H., Calfee C. S., Looney M. R. (2018). Maladaptive role of neutrophil extracellular traps in pathogen-induced lung injury. *JCI Insight*.

[131] Tanaka K., Koike Y., Shimura T. (2014). In vivo characterization of neutrophil extracellular traps in various organs of a murine sepsis model. *PLoS One*.

[132] Chen L., Zhao Y., Lai D. (2018). Neutrophil extracellular traps promote macrophage pyroptosis in sepsis. *Cell Death & Disease*.

[133] McDonald B., Davis R. P., Kim S. J. (2017). Platelets and neutrophil extracellular traps collaborate to promote intravascular coagulation during sepsis in mice. *Blood*.

[134] Biron B. M., Chung C. S., O'Brien X. M., Chen Y., Reichner J. S., Ayala A. (2017). Cl-Amidine prevents histone 3 citrullination and neutrophil extracellular trap formation, and improves survival in a murine sepsis model. *Journal of Innate Immunity*.

[135] McDonald B., Urrutia R., Yipp B. G., Jenne C. N., Kubes P. (2012). Intravascular neutrophil extracellular traps capture bacteria from the bloodstream during sepsis. *Cell Host & Microbe*.

[136] Meng W., Paunel-Görgülü A., Flohé S. (2012). Depletion of neutrophil extracellular traps in vivo results in hypersusceptibility to polymicrobial sepsis in mice. *Critical Care*.

[137] Czaikoski P. G., Mota J. M. S. C., Nascimento D. C. (2016). Neutrophil extracellular traps induce organ damage during experimental and clinical sepsis. *PLoS One*.

